# Using Machine Learning to Predict Resilience Among Nurses in a South African Setting

**DOI:** 10.3390/ijerph22070996

**Published:** 2025-06-24

**Authors:** Jennifer Chipps, Amanda Cromhout, Umit Tokac

**Affiliations:** 1 School of Nursing, Faculty of Community Health Sciences, University of Western Cape, Cape Town 7441, South Africa; 6403233@myuwc.ac.za; 2 College of Nursing, University of Missouri, St. Louis, MO 63121, USA; tokacu@umsl.edu

**Keywords:** nurses, resilience, mental health, machine learning, random forest

## Abstract

Nursing is a stressful profession. Stress can affect the mental health of nurses. A positive response to stress, resilience, is known to be a protective factor against mental health issues. This study aimed to use machine learning with secondary data from five survey studies, conducted between 2022 and 2023, to identify factors predicting high versus low levels of resilience in South African nursing samples from the Western Cape Province, South Africa. The sample included (1134 records (male = 250, 22.0%, female = 874, 77.1%, and other = 10 (0.9%) included all data on all categories of nursing staff (student nurses (567, 50%), professional registered nurses (315, 27.8%), and non-professional nurses (246, 21.7%) who completed a survey using a response to stress scale. We used random forest analysis, demographic variables, years of experience, and a brief 4-item screen of resilience to predict resilience. The model yielded limited added value from demographic groupings in this model, but the brief screening had an overall classification accuracy of 86.41% (95% CI: 0.810; 0.908).

## 1. Introduction

Globally, nursing is a stressful profession characterized by exposure to the emotional impact of death and suffering, staff shortages, long working hours, and limited resources [1]. Evidence has shown that stress experienced at work may result in fatigue and burnout, reduced work and life satisfaction, disruptions in work–life balance, high turnover intention, and mental health problems, such as anxiety and depression [1,2,3,4].

A positive response to this stress, or resilience, is a protective factor against negative psychological outcomes [5]. Nurses with higher levels of resilience are better able to respond to stress in the clinical setting and generally report higher levels of mental well-being, which also ensures better patient outcomes [6]. Resilience can be described as a multifaceted and evolving process that, when maintained, supports positive adaptation to workplace stress, protects against psychological harm, and promotes the continued delivery of safe, high-quality patient care [7]. In this study, based on the work of Bonano [8], resilience was conceptualized as a response to stressful events, with cognitive strategies such as active coping, cognitive flexibility, meaning-making, self-efficacy, and spirituality being positive responses to stress [9].

To enable targeted support strategies such as active coping and cognitive reframing skill development [7], it is important to identify factors and tools which can predict low and high resilience in nurses. However, findings across studies have reported mixed results in terms of socio-demographic variables such as sex, age, work experience, education, and shift work nurse significantly predicting resilience [10].

With the emergence of machine learning and the ability to analyse large datasets, it is possible to uncover insights into the prediction of resilience that may not be available with traditional statistical methods [11,12]. One study, which used random forest, support vector machines, and backpropagation artificial neural networks, investigated factors contributing to academic resilience in nursing and reported accuracies ranging from 73.0% to 76.9% [12]. Similarly, a South African study, performed to predict risk factors for burnout and emotional exhaustion in nursing staff, reported accuracies of 75.8% (feelings of burnout) and 76.8% (emotional exhaustion) for the gradient boost classifier (GBC), and accuracies of 64.4% (feelings of burnout) and 68.5% (emotional exhaustion) using demographic data [13].

## 2. The Present Study

This study aimed to use random forest machine learning analysis to investigate factors that could be used to predict high versus low levels of resilience in South African nursing staff from the Western Cape Province, South Africa. Random Forest was deemed most suitable due to its demonstrated ability to model the complex, non-linear relationships that are often present in health science survey responses and its utility in ranking feature importance [14].

## 3. Materials and Methods

### 3.1. Setting and Sample

The primary data were extracted from five survey studies conducted in the metropolitan area in Cape Town, Western Cape Province, South Africa between 2022 and 2023. The sample included 1134 records (male = 250, 22.0%, female = 874, 77.1%, other = 10, 0.9%) and included all nursing staff (student nurses from two universities offering nursing education (567, 50%), professional nurses (315, 27.8%), and non-professional nurses (246, 21.7%).

### 3.2. Predictors and Outcome Metric

The outcome metric for the study was high or low resilience, determined based on the total score on the Response to Stress Scale (RSES-22) [9]. The scale is specifically focused on an individual’s characteristic responses to stressful life events [9]. The scale is a 22-item self-report measure, evaluating the cognitive, emotional, and behavioral responses to stressful life events on a Likert scale ranging from 0 to 4. The scale has five factors, namely, meaning making and restoration, active coping, cognitive flexibility, spirituality, and self-efficacy behaviors [9]. The scale had sufficient reported internal consistency reliability (α = 0.91–0.93), good test–retest reliability (r = 0.87) and convergent, divergent, and concurrent validity [9]. Resilience was categorized as low (low to moderate scores below 70) or high (scores between 71 and 88) based on the total score. In addition, a brief 4-item scale, the Response to Stress Scale (RSES-4) [15], was included to screen for resilience. This screen is based on four (4) items from the RSES-22, one item from each of the meaning-making, active coping, cognitive flexibility, and self-efficacy factors of the RSES-22 [15]. This brief scale had previously reported comparable internal consistency reliability and test–retest reliability with the RSES-22 [15].

The available proposed predicator variables included in the analysis were the demographic variables of age and gender; the professional variables of years of experience as a nurse or nursing student, stages of professional development (student nurse, novice nurse, moderately experienced nurse, or experienced or veteran nurse), and juniority (being a student nurse); and a brief screen, the RSES-4. The RSES-4 was included due to evidence that when measuring psychological constructs, shorter scales are useful as they are time- and cost-effective and are not as burdensome for participants, who may feel fatigued and unmotivated when completing longer scales [15]. These brief scales would enable routine checks of resilience via innovative integration into AI solutions, such as chatbots.

### 3.3. Statistical Analysis

Secondary data analysis was conducted, and a random forest estimation method was used to predict resilience among the respondents. Random forest is a machine learning algorithm that combines multiple decision trees to produce a more accurate prediction. In this random forest model, the ensemble consisted of 500 decision trees. Each tree was constructed by considering two randomly selected features at each split point. To prevent overfitting and ensure the robustness of individual trees, a minimum of 20 observations were required in a node for it to be split further. These hyperparameter choices aim to balance the model’s ability to capture complex relationships within the data while maintaining generalization performance and preventing excessive variance. Random forest was implemented using the R programming language, version 4.0.3, and the R package (4.3.3) random forest [16]. Datasets were divided into training and testing sets and a 80:20 split was used, with 80% of data used for training and 20% for testing [14] (Table 1). The training set was used to develop the random forest model, while the testing set was used to evaluate the performance of the model.

The random forest model predicted high or low resilience among nurses for each respondent in the testing set. Resilience was classified into two categories, namely ‘resilient’ or ‘less resilient’, based on the RSES-22 score, with scores between 71 and 88 indicating ‘resilient’ and 0 and 70 indicating ‘less resilient’ [15]. The predicted resilience category was compared to the actual resilience category to evaluate the accuracy of the model and the use of the following performance metrics to assess the model’s performance: sensitivity, specificity, positive predictive value, and negative predictive value. In addition, to include predictors with the highest importance in the model, mean decrease accuracy values were calculated. The mean decrease in accuracy is calculated to monitor the impact of each predictor on the accuracy of a random forest model and is a feature-of-importance measure used in random forest models to assess the impact of each predictor variable on the model’s accuracy. This measure reflects the decrease in model accuracy when a particular variable is randomly permuted while all others are left unchanged.

The mean decrease in accuracy is computed by comparing the out-of-bag (OOB) error rate of the original model to the OOB error rate after permuting each predictor variable. Variables that result in larger decreases in accuracy when permuted are considered more important. A feature importance plot ranks variables based on their importance to a random forest model, with higher values indicating greater importance. This method provides insight into how each variable contributes to a model’s overall predictive performance, considering both the variable’s individual effect and its interactions with other variables in the model.

### 3.4. Ethical Considerations

The primary studies received ethical approval from the university ethics committees. 

## 4. Results

### 4.1. Demographics

The data included 874 females (77.1%), with the sample having an average age of 31.5 years (ranging from 18–64 years). In terms of stages of professional development, the data included 567 student nurses (50%), 70 (6.2%) novice nurses (0–2 years’ experience), 113 (10.0%) early-career nurses (3–5 years’ experience), 245 (21.6%) mid-career nurses (6–15 years), 78 (6.9%) experienced nurses (16–25 years’ experience), and 54 (4.8%) veterans (26+ years’ experience). Based on the RSES-22 scale, the average score was 69.8 (±15.8), with 519 (45.7%) classified as ‘less resilient’ and 614 (54.1%) as ‘resilient’. The average score of RSES-4 was 13.1 (±2.9) (ranging from 0 to 16) (Table 2).

### 4.2. Key Predictors

A random forest classifier was trained to predict participants’ resilience category (resilient’ vs. less resilient’) based on predictors including RSES-4 scores, experience, age, experience group, student status (student vs. non-student), and gender.

**Figure 1:**
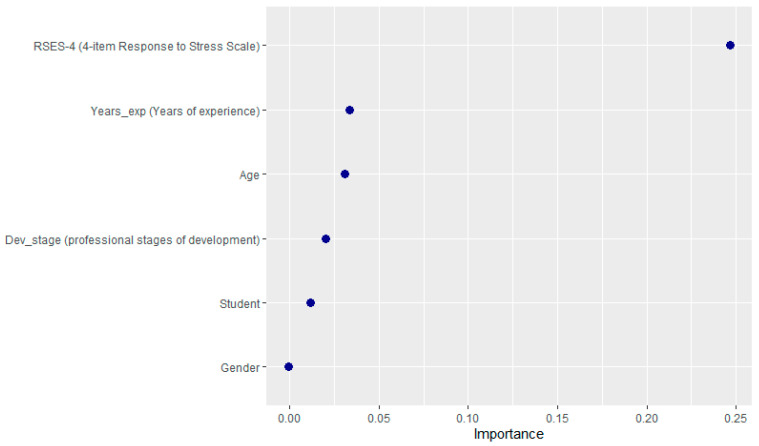
Feature importance.

The brief scale (RSES-4) was the most important predictor in the model, followed by years of experience in the role (Figure 1). The variable importance plot, based on the mean decrease in accuracy, indicates the relative contribution of each predictor in classifying participants’ resilience categories (resilient vs. less resilient). The brief scale emerged as the most influential predictor (importance ~0.25), suggesting that executive functioning (as measured by RSES-4) plays a critical role in distinguishing resilience levels. Years of experience and age followed in importance (~0.15–0.20), implying that accumulated experience and developmental stage also significantly impact resilience classification. In contrast, the experience group, student status, and gender showed minimal importance (all ≤ 0.05), indicating negligible effects on model accuracy.

### 4.3. Accuracy

The overall classification accuracy of the random forest model was 86.41%, with a 95% confidence interval (CI) of [0.810, 0.908], significantly exceeding the no-information rate (53.88%), *p* < 0.001. The confidence interval signifies the range within which the true overall accuracy of the model, if applied to the entire population from which our data was sampled, is likely to fall. More precisely, it means that if we were to repeatedly sample data and build our model using the same methodology, the 95% of the confidence intervals constructed from those experiments would contain the true population accuracy. The relatively narrow width of this interval suggests a reasonably precise estimate of the model’s performance.

**Figure 2:**
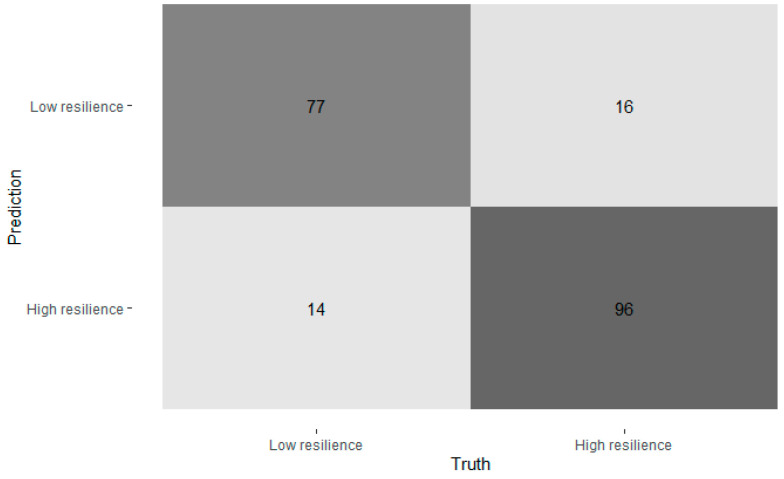
Confusion matrix of predicted vs. actual resilience categories.

The confusion matrix (Figure 2) indicated that the model was correctly classified: there were 77 (true positives) out of 95 ‘less resilient’ cases (sensitivity = 83.16%) and 99 (true negatives) out of 111 ‘resilient’ cases (specificity = 89.19%). The positive predictive value (PPV) was 86.81%, indicating that when the model predicted low resilience, it was correct 86.81% of the time. The negative predictive value (NPV) was 86.09%, suggesting that high-resilience predictions were accurate 86.09% of the time. Cohen’s kappa (κ = 0.726) indicated substantial agreement between predicted and observed classifications. McNemar’s test for symmetry was non-significant (*p* = 0.571), suggesting there was no significant bias in misclassifications. The balanced accuracy (mean of sensitivity and specificity) was 86.17%, further supporting the model’s robust performance. These results suggest that the random forest model effectively distinguishes between low and high resilience based on the given predictors, with particularly strong specificity and a high overall classification rate.

### 4.4. Random Forest Classification Predicting Resilience Levels

The model achieved an overall classification accuracy of 86.41% (95% CI [0.810, 0.908]), significantly exceeding the no-information rate (53.88%), *p* < 0.001. The confusion matrix indicated that the model correctly classified:77 (true positives) out of 95 low-resilience cases (sensitivity = 83.16%) and 99 (true negatives) out of 111 high-resilience cases (specificity = 89.19%). The positive predictive value (PPV) was 86.81%, indicating that when the model predicted low resilience, it was correct 86.81% of the time. The negative predictive value (NPV) was 86.09%, suggesting that high-resilience predictions were accurate 86.09% of the time. Cohen’s kappa (κ = 0.726) indicated substantial agreement between predicted and observed classifications. McNemar’s test for symmetry was non-significant (*p* = 0.571), suggesting no significant bias in misclassifications. The balanced accuracy (mean of sensitivity and specificity) was 86.17%, further supporting the model’s robust performance. These results suggest that the random forest model effectively distinguishes between low and high resilience based on the predictors given, with particularly strong specificity and a high overall classification rate (Table 3).

## 5. Discussion

Applying random forest analysis, our results indicated that the RSES-4 and years of experience were the most important predictors of resilience in this respondent group, with the model showing high accuracy regarding predicting the ‘less resilient’ and ‘resilient’ groups. Specifically, belonging to one of these categories was correctly predicted 86.81% and 86.09% of the time, respectively, with an overall classification accuracy of 86.41% (95% CI [0.810, 0.908]). The results are in line with previous research that showed high accuracy in predicting resilience and other indicators of well-being in nursing samples [12,13]. Similar to the study in South Africa by Van Zyl-Cillie [13], our study confirms the limitations of predicting resilience from demographic data alone [13] and adds to the research displaying conflicting outcomes with regard to the association between socio-demographic variables [10,17], emphasizing cognitive and experiential factors over demographic variables in resilience prediction.

The key finding about the predictive value of a brief scale, the RSES-4, in our study is particularly important for supporting nursing staff. Firstly, the RSES-4 appeared to have high sensitivity, supporting its use as a brief measure of resilience, and showed similar performance to the longer RSES-22, with internal consistency (α = 0.76–0.78), test–retest reliability, and criterion validity [15]. Secondly, out findings point towards its use in identifying individuals in need of support. The findings are in line with previous research where the RSES-4 was found to be a valid and reliable brief screen among different categories of workers such as law enforcement, medical emergency treatment, and fire rescue [18]. The RSES-4 thus shows specific potential for future use as a brief measure of resilience among nurses. In the high-stress and heavy- workload setting of nursing, brief measures are particularly useful as they are time- and cost-effective and do not interrupt the workflow, allowing for quick assessments and interventions [10,19]. Answering long questionnaires can increase the workload, and it is useful to have brief, valid measures which can be followed if required instead of longer measures [20]. Screening can then be the first step in tailored resilience training programmes to improve coping, stress regulation, and job satisfaction in nurses [21]. The RSES-4 also shows potential for incorporation into digital applications, such as chatbots, where shorter assessment tools are desirable [15]. Using chatbots for on-demand screening and personalized interventions can be the new frontier in supporting nurses [22], providing screening and links to validated chatbots for support such as Wysa [23].

The second key finding, the association between experience and resilience, is not new, with several studies reporting correlations between resilience and years of experience and age in nurses [5,24,25,26]. A meta-synthesis found that nurses regarded their years of work experience as contributing to their ability to deal with stress in the workplace [24]. With more years of experience, nurses report increased self-efficacy [25,27], which is the ability to handle difficult situations or stress [27]. Experienced nurses have typically been more exposed to complex situations, had more training, gained more practical knowledge, and had more opportunities to learn from mistakes [28,29]. This results in increased confidence related to accumulated knowledge, practiced skills, and success in previously responding positively to stress [27]. Exposing nurses to opportunities to develop self-efficacy can increase resilience, as was reported in a study of student nurses in South Africa during COVID-19, where student nurses returning to clinical placement were exposed to a psychological first aid programme [29,30].

Lastly, as no routine dataset exists in this field, this study was limited in that it was based on the secondary analysis of a number of surveys, similar to the study by Van Zyl-Cillie [13].

## 6. Conclusions

Machine learning can provide insights that may not be available with traditional statistical methods [11] with relatively high accuracy. Our findings provide evidence that years of experience (as a proxy for self-efficacy) and the use of a brief scale (the RSES-4) are predictors of resilience. The findings support research that showed that experienced nurses are better prepared to cope with the challenges of the nursing profession due to their exposure and greater self-efficacy and point towards the utility of brief measures, such as the RSES-4, for resilience screening. Brief measures are particularly useful in clinical and digital contexts (e.g., chatbots), where short screening tools provide time- and cost-efficient means of assessment, especially when early intervention is crucial.

## Figures and Tables

**Table 1:** Train and test datasets.

	Train	Test
‘Resilient’ (71–88)	445	109
‘Less resilient’ (0–70)	378	93

**Table 2:** Demographic and well-being profile of sample.

Variable	Value
Age	
Mean age	31.5 years
Range	18–64 years
Gender	
Female	874 (77.1%)
Male	250 (22.0%)
Other	10 (0.9%)
Stages of professional development	
Student nurses	557 (49.1%)
Novice nurses (0–2 years’ experience)	70 (6.2%)
Early-career nurses (3–5 years’ experience)	113 (10.0%)
Mid-career nurses (6–15 years’ experience)	245 (21.6%)
Experienced nurses (16–25 years’ experience)	78 (6.9%)
Veterans (26+ years’ experience)	54 (4.8%)
RSES-22 (0–88)	
Mean, SD	69.8 (±15.8)
‘Less resilient’ (0–70)	519 (45.7%)
‘Resilient’ (71–88)	614 (54.1%)
RSES-4 (0–16)	
Mean, SD	13.1 (±2.9)

**Table 3:** Performance metrics of the random forest model for predicting resilience in group members.

Metric	Value	95% Confidence Interval	*p*-Value
Overall Accuracy	86.41%	[0.810, 0.908]	<0.001
No-Information Rate	53.88%	—	—
Sensitivity (True Positive Rate)	83.16% (79/95)	—	—
Specificity (True Negative Rate)	89.19% (99/111)	—	—
Positive Predictive Value (PPV)	86.81%	—	—
Negative Predictive Value (NPV)	86.09%	—	—
Cohen’s Kappa (κ)	0.726	—	—
McNemar’s Test (Symmetry)	—	—	0.571
Balanced Accuracy	86.17%	—	—

## Data Availability

Data is unavailable due to privacy or ethical restrictions.
